# Prevalence and risk factors related to preterm birth in Brazil

**DOI:** 10.1186/s12978-016-0230-0

**Published:** 2016-10-17

**Authors:** Maria do Carmo Leal, Ana Paula Esteves-Pereira, Marcos Nakamura-Pereira, Jacqueline Alves Torres, Mariza Theme-Filha, Rosa Maria Soares Madeira Domingues, Marcos Augusto Bastos Dias, Maria Elizabeth Moreira, Silvana Granado Gama

**Affiliations:** 1 National School of Public Health - Oswaldo Cruz Foundation, Rua Leopoldo Bulhões, 1480, sala 809, Manguinhos, Rio de Janeiro, CEP 21041-210 Brazil; 2 National Institute of Women, Children and Adolescents Health Fernandes Figueira, Oswaldo Cruz Foundation, Avenida Rui Barbosa 716 - Flamengo, Rio de Janeiro, RJ Brazil; 3 National Health Agency - Ministry of Health, Av. Augusto Severo, 84 - Glória, Rio de Janeiro, RJ 20021-040 Brazil; 4 National Institute of Infectious Disease, Oswaldo Cruz Foundation, Avenida Brasil 4.365 - Manguinhos, Rio de Janeiro, RJ CEP 21040-900 Brazil

**Keywords:** Spontaneous preterm birth, pPROM, Provider-initiated preterm birth, Risk factors, Brazil

## Abstract

**Background:**

The rate of preterm birth has been increasing worldwide, including in Brazil. This constitutes a significant public health challenge because of the higher levels of morbidity and mortality and long-term health effects associated with preterm birth. This study describes and quantifies factors affecting spontaneous and provider-initiated preterm birth in Brazil.

**Methods:**

Data are from the 2011–2012 “Birth in Brazil” study, which used a national population-based sample of 23,940 women. We analyzed the variables following a three-level hierarchical methodology. For each level, we performed non-conditional multiple logistic regression for both spontaneous and provider-initiated preterm birth.

**Results:**

The rate of preterm birth was 11.5 %﻿, (95 % confidence 10.3 % to 12.9 %) 60.7 % spontaneous - with spontaneous onset of labor or premature preterm rupture of membranes - and 39.3 % provider-initiated, with more than 90 % of the last group being pre-labor cesarean deliveries. Socio-demographic factors associated with spontaneous preterm birth were adolescent pregnancy, low total years of schooling, and inadequate prenatal care. Other risk factors were previous preterm birth (OR 3.74; 95 % CI 2.92–4.79), multiple pregnancy (OR 16.42; 95 % CI 10.56–25.53), abruptio placentae (OR 2.38; 95 % CI 1.27–4.47) and infections (OR 4.89; 95 % CI 1.72–13.88). In contrast, provider-initiated preterm birth was associated with private childbirth healthcare (OR 1.47; 95 % CI 1.09–1.97), advanced-age pregnancy (OR 1.27; 95 % CI 1.01–1.59), two or more prior cesarean deliveries (OR 1.64; 95 % CI 1.19–2.26), multiple pregnancy (OR 20.29; 95 % CI 12.58–32.72) and any maternal or fetal pathology (OR 6.84; 95 % CI 5.56–8.42).

**Conclusion:**

The high proportion of provider-initiated preterm birth and its association with prior cesarean deliveries and all of the studied maternal/fetal pathologies suggest that a reduction of this type of prematurity may be possible. The association of spontaneous preterm birth with socially-disadvantaged groups reaffirms that the reduction of social and health inequalities should continue to be a national priority.

## Background

Preterm birth is the largest risk factor for infant morbidity and mortality, not only in the immediate neonatal period but also in infancy, childhood, and even adulthood [1]. It can affect physical health, cognitive and behavioral dimensions, making it one of the most significant challenges for modern public health [2].

Preterm birth can be subgrouped as extreme (less than 28 weeks), severe (between 28 and 32 weeks), and moderate or “near-term” (32 to 36 weeks). In 2005, a committee of experts organized by the National Institute of Child Health and Human Development of the National Institutes of Health (NIH) in the USA suggested the term “late preterm” for newborns with gestational age (GA) between 34 0/7 and 36 6/7 weeks [3, 4]. Concerning its determining factor, preterm birth is classified as either spontaneous, by preterm premature rupture of membranes (pPROM) or provider-initiated, when provoked by medical intervention via induction or pre-labor cesarean section [5].

Recent decades have seen a great increase in the survival of preterm infants, linked to technological advances in neonatal intensive care. The rate of preterm birth has also increased worldwide, largely driven by increases in late preterm birth, often associated with obstetric interventions designed to reduce maternal and fetal complications [3, 6, 7]. In the USA alone, more than half a million preterm births occur each year, making preterm birth an important national public health problem [8].

Brazil has a National Information System on Live Births (SINASC) that gather secondary data about GA at birth, therefore allowing the estimation of the prematurity rate for the country. However, this system still fails to provide a reliable GA estimate. Until 2010, GA at birth was collected in broad intervals of gestational weeks, and the prematurity rate was much underestimated (of 7.1 %) with local studies [9, 10] finding much higher values than those reported by SINASC. From 2011, although SINASC started to collect GA as a continuous variable, the method of estimation changed to rely mainly upon the last menstrual period (LMP) - a method that was previously reported as unsuitable within the Brazilian context [9]. *Birth in Brazil* was the first Nationwide Perinatal Survey in the country, which allowed the analysis on the relationship between preterm births and obstetric interventions, especially the effect of cesarean sections on the rate of preterm births, using primary-data. Brazil has the world’s highest rate of cesarean section - 57 % of all live births and nearly 90 % among women receiving private healthcare at childbirth, in 2013 [11] - suggesting that many of them are for non-medical reasons. Leal et al. [12] found that 45 % of low-risk mothers without obstetric complications, who gave birth to healthy infants, had cesarean sections.

This study aimed to describe the rate of preterm birth in Brazil by geographic region, childbirth healthcare provision (public or private), subgroups of gestational age and determining factor and to investigate risk factors for spontaneous/pPROM and provider-initiated preterm birth.

## Methods

### Data sources

The “Birth in Brazil” study was a national population-based study of postpartum women and their newborns, carried out from February 2011 to October 2012. It recruited a complex sample of 266 hospitals, with 90 women interviewed in each hospital and a total of 23,940 puerperal women and 24,061 live births. Data were weighted by the inverse of the probability of inclusion of each puerperal woman in the sample. A calibration procedure was used to ensure that the distribution of puerperal women sampled was similar to that observed among the population for the year 2011. Further information on the design of the sample is detailed elsewhere [13]. All women who had given birth to a live newborn, regardless of weight or GA, or had a stillbirth (with birth weight ≥ 500 g and/or GA ≥ 22 weeks) in one of the sampled hospitals during the data collection period were invited to participate. Face-to-face interviews were held with the postpartum women during their hospital stay, and data about the women and their newborns were collected from their medical records, and extracted from photographs of prenatal care cards. Women and newborns who remained as inpatients, including those transferred to other hospitals, were tracked by the study for as long as 28 days (for newborns) and 42 days (for women). More details on the data collection have been published elsewhere [14].

### Subjects

**Fig. 1:**
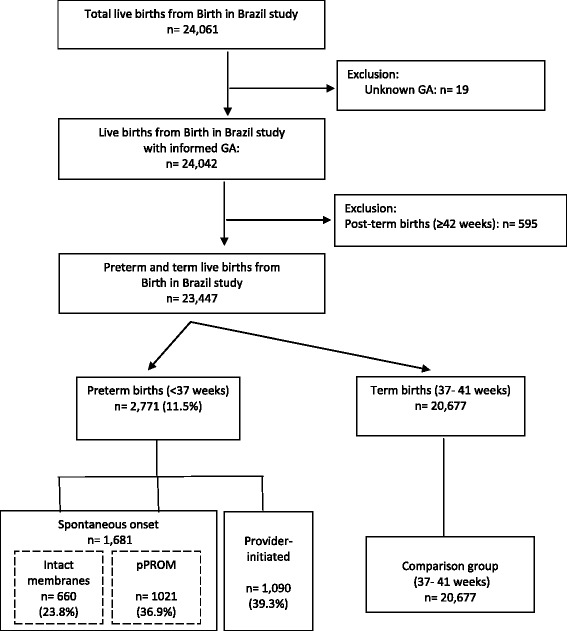
Flowchart of final sample

In the current analysis, we included all preterm and term live births from the “Birth in Brazil” study, defined as having a GA at birth of <37 weeks and from 37^0/7^ to 41^6/7^ weeks, respectively. We excluded 19 newborns with undetermined GA, and 595 post-term newborns (GA of 42 weeks or more). Our final sample was 2,771 premature newborns and 20,677 term newborns, as shown in Fig. 1. GA was calculated by an algorithm that primarily relied upon early ultrasound estimates [15].

### Outcome variable

The outcome was the incidence of prematurity, which we categorized as spontaneous/pPROM or provider-initiated. Spontaneous/pPROM preterm births were either with spontaneous onset of labour or with preterm premature rupture of membranes. Provider-initiated preterm births were initiated by labor induction or pre-labor cesarean section. Women with pre labor rupture of membranes who gave birth through labor induction or pre labor cesarean were also classified as spontaneous/pPROM births. We classified as induction of labor women with intact membranes who received medical intervention to initiate uterine contraction before a spontaneous onset of labor, irrespective of whether the type of delivery was vaginal or cesarean. Pre-labor cesarean sections were the sections with no spontaneous or induction of labor.

### Exposure variables and definitions

We evaluated the classical risk factors for prematurity including socioeconomic and demographic factors, previous preterm birth, smoking during pregnancy, pre-pregnancy body mass index (BMI), multiple pregnancy, and maternal/fetal conditions during pregnancy. We also evaluated factors that we suspected may play a role in the Brazilian context, including source of payment for childbirth healthcare, number of previous cesarean sections, and adequacy of prenatal care. We organized the variables using a hierarchical model proposed by Victora (1997) [16]. For the first level, we considered the socioeconomic and demographic variables: “childbirth healthcare provision” (public or private); “age” (12 to 19, 20 to 34, or 35 years and over); “self-reported skin color” (white, black, or brown); “marital status” (living with partner or not); and “years of schooling” (7 or less, 8 to 10, 11 to 14, or 15 or more years). For the second level, we considered obstetric history variables: “parity” (nulliparous, 1–2, ≥3); “previous abortion” (yes, no); “previous stillbirth or neonatal death” (yes, no); “previous preterm birth” (yes, no); and “number of previous cesarean sections (none, one, two or more). For the third level, we considered maternal/fetal care and conditions during pregnancy: “prenatal care” (adequate-plus/adequate or inadequate/partially adequate); “pre-pregnancy body mass index (BMI)” (≤18.5, 18.5–24.9, 25.0–29.9, or ≥30); “smoking during the third trimester of pregnancy” (no, <10 cigarettes/day, or ≥10 cigarettes/day); “type of pregnancy” (single, multiple); and the following pathologies: hypertensive disorders (chronic hypertension, pre-eclampsia and HELLP syndrome); eclampsia, pre-existing diabetes, gestational diabetes; severe chronic diseases; infection at hospital admission for birth (including urinary tract infection and other sever infection such as chorioamnionitis and pneumonia); placental abruption; placenta previa; intrauterine growth restriction (IUGR) and major newborn malformation (including anencephaly, hydrocephaly, spina bifida, gastrosquisis and other abdominal wall defects, cardiac malformations and multiple malformations). Women with the presence of any of these conditions were considered “high obstetric risk” and others were “low obstetric risk”. Age, pre-pregnancy BMI, skin color, marital status, years of schooling, and smoking status were reported by the women at the time of interview. All other variables were collected from the patient’s medical records. For approximately 20 % of puerperal women (4,763), pre-pregnancy BMI data were imputed via Sequential Regression Trees using the MICE (Multivariate Imputation by Chained Equations) package from R Project software.

Women who gave birth in public or mixed-funding hospitals (but who were not covered by private health insurance plans) were considered to have received “public childbirth healthcare”. Women whose birth was covered by a private health insurance plan, and those who gave birth in private facilities regardless of coverage by a health insurance plan, were considered to have received “private childbirth healthcare”.

Adequacy of prenatal care was assessed using an adapted version of the Kotelchuck Index [17]. This measures adequacy of prenatal care by two independent and distinct dimensions: initiation, and once service provision has begun. The expected number of visits was based on the Brazilian Ministry of Health’s recommendations [18] (of a minimum of six visits: one in the first trimester, two in the second and three in the third) and adjusted for the GA at initiation of care and at delivery. The measure for adequacy of received services is the ratio between the actual and expected number of visits. The dimensions were combined into a single summary prenatal care utilization index, with four categories. Inadequate care was defined as either prenatal care initiated after the 12th week of gestation or fewer than 50 % of the recommended visits being completed. All other categories required initiation of care between the beginning of pregnancy and before the 12th week of gestation: intermediate (50–79 % of recommended visits), adequate (80–109 %), and adequate-plus (more than 110 %) [18, 19]. We grouped “adequate-plus” with “adequate,” and “intermediate” with “inadequate,” to create a dichotomous variable.

### Statistical analysis

The rate of preterm birth was calculated by dividing the number of live births before 37 weeks of gestation by the total number of live births. Differences in this rate were assessed by 95 % confidence intervals (CI), taking into account the sample weights and the clustering design. Differences in proportions of maternal characteristics for spontaneous/pPROM and provider-initiated preterm births, compared with those for term births, were analyzed by chi-square statistical tests with a significance level of <0.05.

We performed non-conditional multiple logistic regressions separately for spontaneous/pPROM and provider-initiated preterm birth. In the first multivariable model (Model A), all first-level variables were included. Those with estimated significance <0.10 were included in the second model, along with all second-level variables (Model B). In the third model, first-level and second-level variables with estimated significance <0.10 were analyzed with the third-level variables. All variables with a significance level <0.05 were retained in the final multivariable model (Model C). The results from all models were expressed as adjusted odds ratios (OR) with their corresponding 95 % CI. In all statistical analyses, the complex sampling design was taken into consideration. The statistical program used for analysis was SPSS, version 20.0 (SPSS Inc., Chicago, USA).

## Results

**Fig. 2:**
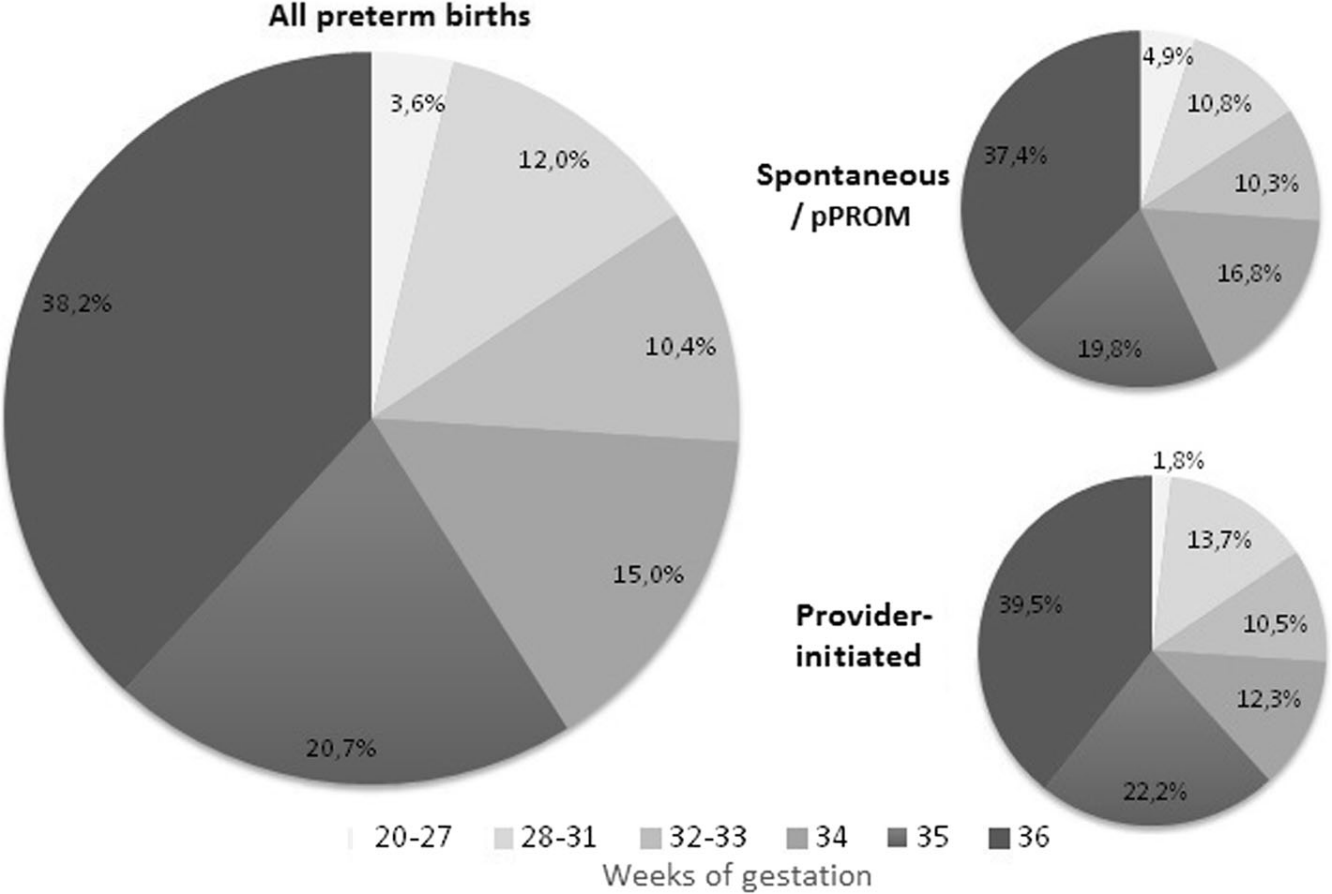
Gestational age distribution according to the determining factor of birth

The overall rate of preterm birth in Brazil for the period 2011–2012 was estimated as 11.5 % (95 % CI 10.3–12.9), without significant differences by broad geographic region or type of childbirth healthcare (public or private), but was slightly higher in state capitals. Late preterm was the largest category, at 74 % of preterms, or 8.5 % of all births. Spontaneous/pPROM preterm births made up 60.7 % of all preterm births, and were because of pPROM in about one-third of cases. There was a higher portion of extremely premature births (5 %) in this group than among provider-initiated preterm births (2 %). Provider-initiated preterm births were 39.3 % of the total, due almost entirely (90 %) to pre-labor cesarean section (Table 1, Fig. 2).

**Table 1:** Preterm delivery according to some demographic characteristics, gestational age and determining factor

	n	%	rate	95 % CI
Total	2,771	100.0	11.5	(10.3–12.9)
Region				
North	300	10.9	13.0	(8.5–19.5)
Northeast	901	32.5	12.9	(10.1–16.4)
Southeast	1,062	38.3	10.4	(8.9–12.1)
South	336	12.1	11.2	(9.6–12.9)
Midwest	172	6.2	11.0	(8.5–14.0)
Locality				
State capital	1,253	45.2	14.0	(11.5–17.0)
Non state capital	1,518	54.8	10.1	(8.9–11.3)
Childbirth healthcare				
Public	2,191	79.1	11.4	(10.1–12.9)
Private	580	20.9	11.9	(9.5–15.0)
Gestational age (weeks) ^a^				
< 28	100	3.6	0.4	(0.3–0.6)
28–31	333	12.0	1.4	(1.0–1.9)
32–33	288	10.4	1.2	(0.9–1.5)
34–36	2,050	74.0	8.5	(7.7–9.4)
Determining factor^a^				
Spontaneous/pPROM	1,681	60.7	7.0	(6.2–7.9)
Intact membranes	660	23.8	2.7	(2.3–3.2)
pPROM	1,021	36.9	4.2	(3.8–4.7)
Provider-initiated	1,090	39.3	4.5	(4.1–5.1)
Labor induction	60	2.2	0.2	(0.1–0.8)
Prelabor Cesarean	1,030	37.2	4.3	(4.0–4.7)

^a^For each category the rate is based on the total number of live births, of 24,042

Compared with term deliveries, spontaneous/pPROM preterm births occurred more frequently in adolescent women, women with lower levels of schooling, nulliparous women, and those with histories of fetal/neonatal demise, a prior premature infant, or no prior cesarean section. Provider-initiated preterm births were associated with private childbirth healthcare, maternal age of 35 or greater, higher levels of schooling, living with a partner, and prior history of abortion, fetal/neonatal demise, a premature infant, and prior cesarean section (Table 2).

**Table 2:** Maternal characteristics of spontaneous/pPROM and provider-initiated preterm births as compared to term births

	Spontaneous/pPROM preterm births		*P*-value of Spontaneous vs. Term	Provider-initiated preterm births		*P*-value of Provider-initiated vs. Term	Term births (37–41 weeks)	
	n	%		n	%		n	%
Total	1,681	100.0		1,090	100.0		20,677	100.0
Level 1 - Sociodemographic characteristics								
Childbirth healthcare								
Public	1,439	85.6	0.065	758	69.5	<0.001	16,445	79.5
Private	241	14.3		332	30.4		4,232	20.5
Age (*n* = 1,678, 1,087 and 20,674)								
12 to 19	462	27.5	<0.001	145	13.4	<0.001	3,840	18.6
20 to 34	1,051	62.6		754	69.4		14,692	71.0
≥ 35	165	9.8		188	17.3		2,142	10.4
Skin color (*n* = 1,681, 1,087 and 20,673)								
White	522	31.1	0.312	414	38.1	0.084	7,075	34.2
Black	155	9.2		70	6.5		1,765	8.5
brown	1,003	59.7		603	55.5		11,833	57.3
Marital status (*n* = 1,678, 1,087 and 20,659)								
Live with a partner	1,356	80.4	0.701	927	85.3	0.024	16,818	81.4
Do not live with a partner	321	19.0		160	14.7		3,841	18.6
Years of schooling (*n* = 1,672, 1,083 and 20,582)								
≤ 7	544	32.5	<0.001	230	21.2	<0.001	5,349	26.0
8 to 10	469	28.0		222	20.5		5,265	25.6
11 to 14	562	33.6		475	43.9		8,097	39.3
≥ 15	97	5.8		156	14.4		1,871	9.1
Level 2 - Obstetric history								
Previous births								
0	872	51.9	<0.001	556	51.0	0.032	9,565	46.3
1 to 2	611	36.4		416	38.1		9,029	43.6
≥ 3	197	11.7		118	10.9		2,083	10.1
Previous abortion^a^ (*n* = 918, 626 and 12,166)								
No	635	69.2	0.206	394	63.0	0.005	8,710	71.6
Yes	283	30.8		232	37.0		3,456	28.4
Previous stillbirth or neonatal death^b^ (*n* = 810, 534 and 11,113)								
No	729	90.1	0.003	468	87.6	<0.001	10,385	93.4
Yes	80	9.9		66	12.4		728	6.6
Previous preterm birth^b^ (*n* = 810, 534 and 11,113)								
No	584	72.1	<0.001	393	73.7	<0.001	9,992	89.9
Yes	226	27.9		140	26.3		1,121	10.1
Previous caesarean section^b^ (*n* = 810, 534 and 11,113)								
No	587	72.5	<0.001	241	45.0	<0.001	6,585	59.2
1	171	21.2		202	37.8		3,465	31.2
≥ 2	51	6.3		91	17.1		1,063	9.6
Level 3 - Maternal/fetal conditions during pregnancy								
Adequacy of antenatal care (*n* = 1,621, 1,061 and 20,175)								
Adequate	952	58.7	<0.001	791	74.5	<0.001	13,327	66.1
Inadequate or partially adequate	669	41.3		270	25.5		6,848	33.9
Pre-pregnancy weight status (BMI) (*n* = 1,666, 1,078 and 20,604)								
Urderweight (≤18,5)	147	8.8	0.100	75	7.0	<0.001	1,656	8.0
Normal weight (18,5-24,9)	1,066	64.0		593	55.0		12,719	61.8
Overweight (25–29,9)	342	20.5		259	24.0		4,326	21.0
Obese (≥30)	112	6.7		152	14.1		1,903	9.2
Smoking during pregnancy								
No	1,553	92.4	0.069	1,050	96.3	0.076	19,484	94.3
Yes, less than 10 cigarettes/day	69	4.1		29	2.6		670	3.2
Yes, 10 or more cigarettes/day	58	3.5		12	1.1		523	2.5
Type of pregnancy								
Single	1,483	88.2	<0.001	924	84.7	<0.001	20,476	99.0
Multiple	198	11.8		166	15.3		201	1.0
Obstetric risk								
Any below	329	19.6	0.104	720	66.0	<0.001	4,532	21.9
Hypertensive disorders^c^	130	7.8	0.112	460	42.2	<0.001	1,992	9.6
Eclampsia	3	0.2	0.063	41	3.8	<0.001	88	0.4
Preexisting Diabetes	16	1.0	0.857	39	3.6	<0.001	194	0.9
Gestational Diabetes	113	6.7	0.115	168	15.4	<0.001	1,657	8.0
Severe chronic diseases^d^	12	0.7	0.925	26	2.4	<0.001	162	0.8
Infection at hospital admission for birth	19	1.2	<0.001	13	1.1	<0.001	49	0.2
Abruptio placentae	33	2.0	0.005	71	6.5	<0.001	187	0.9
Placental praevia	8	0.5	0.423	25	2.3	<0.001	77	0.4
IUGR	51	3.1	0.020	217	19.9	<0.001	982	4.7
Major newborn malformation^e^	10	0.6	0.025	10	0.9	<0.001	31	0.1

^a^Excluded primigravida women from the analysis

^b^Excluded nuliparous women from the analysis

^c^Chronic hypertension, pre-eclampsia and HELLP syndrome

^d^Chronic renal diseases, chronic cardiac diseases and auto-immune diseases

^e^Included anencephaly, hidrocephaly, spina bifida, gastrosquisis and other abdominal wall defects, cardiac malformations and multiple malformations

Spontaneous/pPROM preterm birth was associated with inadequate prenatal care, multiple pregnancy, infection at hospital admission for birth, abruptio placentae, IUGR, and major newborn malformation. Provider-initiated preterm birth was more frequent in mothers with adequate prenatal care, elevated pre-gestational BMI, and in pregnancies with gestational or fetal pathology (Table 2).

In the adjusted analysis, only maternal age less than 20 and low levels of schooling were associated with spontaneous/pPROM preterm birth and remained in the model into the final analysis (Table 3). Of the intermediate variables, nulliparity, history of prior preterm birth, and lack of prior cesarean section were associated. Finally, of the proximal variables, inadequate prenatal care, multiple pregnancy, infection at hospital admission for birth, and abruptio placentae were associated (Table 3).

**Table 3:** Maternal characteristics associated with spontaneous/pPROM preterm births

	Model A			Model B			Model C		
	OR adj.^d^ (95 % CI)			OR adj.^e^ (95 % CI)			OR adj.^f^ (95 % CI)		
Level 1 - Sociodemographic characteristics									
Childbirth healthcare (ref: public)									
Private	0.82	0.47	1.42						
Age (ref: 20 to 34)									
12 to 19	1.52*	1.29	1.80	1.41*	1.12	1.77	1.37	1.10	1.71
≥ 35	1.10	0.85	1.43	1.13	0.88	1.45	1.06	0.83	1.36
Skin color (ref: white)									
Black	1.02	0.78	1.33						
brown	1.03	0.81	1.33						
Marital status (ref: live with partner)									
Do not live with partner	0.93	0.77	1.12						
Years of schooling (ref: ≥ 15)									
≤ 7	1.49*	0.95	2.33	1.67*	1.16	2.42	1.73	1.16	2.57
8 to 10	1.31	0.83	2.06	1.45*	1.03	2.04	1.50	1.05	2.14
11 to 14	1.17	0.75	1.80	1.24	0.88	1.74	1.29	0.92	1.81
Level 2 - Obstetric history									
Previous births (ref: 1 to 2) ^a^									
0				1.29*	1.06	1.57	1.37	1.15	1.65
≥ 3				1.25*	0.99	1.59	1.21	0.95	1.54
Previous abortion^b^ (ref: no, in women with a previous pregnancy)				0.99	0.80	1.23			
Previous stillbirth or neonatal death^c^ (ref: no, in women with a previous delivery)				1.05	0.76	1.45			
Previous preterm birth^c^ (ref: no, in women with a previous delivery)				3.42*	2.70	4.32	3.74	2.92	4.79
Previous caesarean section^c^ (ref: no, in women with a previous delivery)									
1				0.59*	0.46	0.76	0.61	0.47	0.78
≥ 2				0.51*	0.35	0.74	0.47	0.31	0.71
Level 3 - Maternal/fetal conditions during pregnancy									
Adequacy of antenatal care (ref: adequate)									
Inadequate or partially adequate							1.29	1.09	1.52
Type of pregnancy (ref: single)									
Multiple							16.42	10.56	25.53
Obstetric risk (ref: no)									
Infection at hospital admission for birth							4.89	1.72	13.88
Abruptio placentae							2.38	1.27	4.47

^a^Separate models B and C, not including the other obstetric history variables, were performed to estimate the OR and 95 % CI for the variable “Previous births”

^b^Primigravida women were allocated in a separate category

^c^Nuliparous women were allocated in a separate category

^d^adjusted for type of payment of birth, age, skin color, marital status and schooling

^e^adjusted for age, schooling, previous births, previous abortion, previous stillbirth or neonatal death, previous preterm births and previous caesaren section

^f^adjusted for age, schooling, previous births, previous preterm births, previous caesaren section, adequacy of antenatal care, type of pregnancy, infection at hospital admission for birth and abruptio placeantae

*P-value lower than 0.10

Maternal age of 35 or more and private childbirth healthcare were significantly associated with provider-initiated preterm birth. Of the intermediate variables, nulliparity, history of prior preterm birth, and two or more prior cesarean sections were associated. Of the proximal variables, multiple pregnancy, and maternal or fetal pathology were significant risk factors (Table 4).

**Table 4:** Maternal characteristics associated with provider-initiated preterm births

	Model A			Model B			Model C		
	OR adj.^g^ (95 % CI)			OR adj.^h^ (95 % CI)			OR adj.^i^ (95 % CI)		
Level 1 - Sociodemographic characteristics									
Childbirth healthcare (ref: public)									
Private	1.36*	0.98	1.87	1.40*	1.07	1.85	1.47	1.09	1.97
Age (ref: 20 to 34)									
12 to 19	0.88	0.69	1.12	0.73	0.55	0.95	0.93	0.68	1.20
≥ 35	1.60*	1.27	2.02	1.65*	1.30	2.08	1.27	1.01	1.59
Skin color (ref: white)									
Black	0.82	0.58	1.16						
brown	1.01	0.84	1.22						
Marital status (ref:live with partner)									
Do not live with partner	0.85	0.67	1.09						
Years of schooling (ref: ≥ 15)									
≤ 7	0.74	0.47	1.17						
8 to 10	0.74	0.46	1.19						
11 to 14	0.91	0.60	1.37						
Level 2 - Obstetric history									
Previous births (ref: 1 to 2) ^a^									
0				1.40*	1.16	1.70	1.26	1.04	1.53
≥ 3				1.23*	0.94	1.61	1.16	0.89	1.52
Previous abortion^b^ (ref: no, in women with a previous pregnancy)				1.14	0.84	1.53			
Previous stillbirth or neonatal death^c^ (ref: no, in women with a previous delivery)				1.39*	0.93	2.09			
Previous preterm birth^c^ (ref: no, in women with a previous delivery)				2.84*	2.18	3.70	3.14	2.39	4.11
Previous caesarean section^c^ (ref: no, in women with a previous delivery)									
1				1.41*	1.06	1.87	1.25	0.96	1.62
≥ 2				1.91*	1.43	2.55	1.64	1.19	2.26
Level 3 - Maternal/fetal conditions during pregnancy									
Type of pregnancy (ref: single)									
Multiple							20.29	12.58	32.72
Obstetric risk (ref: no)									
Any below							6.84	5.56	8.42
Hypertensive disorders^d^							6.35	4.97	8.12
Eclampsia							8.67	5.24	14.35
Preexisting Diabetes							3.60	2.19	5.91
Gestational Diabetes							2.04	1.58	2.63
Severe chronic diseases^e^							3.30	1.80	6.05
Infection at hospital admission for birth							5.12	1.76	14.89
Abruptio placentae							7.84	5.04	12.21
Placental praevia							6.44	3.48	11.90
IUGR							5.18	3.93	6.83
Major newborn malformation^f^							5.55	2.00	15.39

^a^Separate models B and C, not including the other obstetric history variables, were performed to estimate OR and 95 % CI for the variable “Previous births”

^b^Primigravida women were alocated in a separate category

^c^Nuliparous women were alocated in a separate category

^d^Chronic hypertension, pre-eclampsia and HELLP syndrome

^e^Chronic renal diseases, chronic cardiac diseases and auto-imune diseases

^f^Included anencephaly, hidrocephaly, spina bifida, gastrosquisis and other abdominal wall defects, cardiac malformations and multiple malformations

^g^adjusted for type of payment of birth, age, skin color, marital status and schooling

^h^adjusted for type of payment of birth, age, previous births, previous abortion, previous stillbirth or neonatal death, previous preterm births and previous caesaren section

^i^adjusted for type of payment of birth, age, previous births, previous preterm births, previous caesaren section, type of pregnancy and obstetric risk

*P-value lower than 0.10

## Discussion

The rate of preterm birth in Brazil in 2011–2012 was high, occurred predominantly as late preterm birth, and was most often spontaneous/pPROM in etiology. It did, however, have a high frequency of initiation by medical intervention, mostly by pre-labor cesarean section, with less than 10 % by induction of labor. Factors that accompany social vulnerability (adolescent pregnancy, low levels of schooling, and inadequate prenatal care) were associated with spontaneous/pPROM preterm birth. Provider-initiated preterm birth was associated with private childbirth healthcare provision and advanced maternal age, which are characteristics commonly related to greater levels of formal employment and higher levels of schooling and income. Pregnancies where the mother had an infection at admission for birth, which are usually subject to identification and early treatment during prenatal care, were at greatest risk of spontaneous/pPROM preterm birth. All of the investigated maternal and fetal pathologies—especially eclampsia and abruptio placentae—were risk factors for provider-initiated preterm birth.

This study has several strengths. First, it is the first to describe preterm birth in Brazil using primary data constituting a representative sample of the entire country. Second, GA was calculated with an algorithm based primarily on early obstetric ultrasound, which confers certain advantages over using date of last menstrual period, as the latter tends to overestimate the rate of preterm birth in the Brazilian population [15]. Third, the classification of the initiation of labor and, consequently, of the type of preterm birth, was carried out by a careful cross-referencing of diverse data in the prenatal and obstetric medical records, increasing internal validity. Nevertheless, it is not without limitations. This study was conducted in institutions where more than 500 deliveries take place each year. It is likely that pregnant women who have a planned or unplanned out of hospital delivery or who deliver in a smaller hospital would have different risks for prematurity. However, given that more than 99 % of deliveries in Brazil take place in hospitals, and approximately 80 % are in larger hospitals, significant changes to the results presented would not be expected. For a small number of women, the GA was estimated by the birth weight (2 %) or by date of last menstrual period (1 %), which may have slightly overestimated the prematurity rate, but is unlikely to have introduced a significant bias to our estimates.

Our results differ slightly from those of a study of 20 public hospitals that are centers of excellence for high-risk obstetrics in Brazil [20]. That found a rate of preterm birth of 12.3 %, with 35 % being provider-initiated. These discrepancies can be attributed to the characteristics of the hospitals, which are public and care for a disproportionately high level of women of lower socioeconomic status. Risk factors for spontaneous preterm birth were found to be comparable to those in our study.

The magnitude of the preterm birth rate in Brazil and the frequency of its determinant factors, both for spontaneous/pPROM and provider-initiated preterm birth, were quite similar to those found in US data, despite large differences in socioeconomic conditions and healthcare systems [1, 21]. The Brazilian rate of preterm birth was nearly twice that found in European countries [22, 23]. Furthermore, among premature, the provider-initiated component corresponded to approximately 40 %; 35 % among women receiving public healthcare at childbirth and 58 % among women receiving private healthcare at childbirth, denoting different models of obstetric care in the country.

Morisaki et al. [24], analyzing risk factors for preterm birth in countries with varying human development indices (HDIs), found an association at the individual level between spontaneous/pPROM preterm birth and lower social conditions for women. The authors were also able to show that the overall preterm birth rates were not related to the HDIs of given nations, but that provider-initiated preterm birth was more frequent in countries with higher indices. Within Brazil, provider-initiated preterm birth was more common in the south east, the region with the greatest HDI in the country (data not shown), and also in state capitals, which have more hospitals that are centers of excellence for the care of high-risk pregnancies and neonates [25].

The excessive medicalization of the management of labor and delivery is regarded as one of the characteristics of the current obstetric care transition in Brazil [26]. With a low fertility rate, a predominance of non-communicable disease, an increase in maternal age, and a moderate burden of maternal mortality, Brazil as an emerging economy has also shown a large and continually rising rate of cesarean sections [27].

The contradiction between higher socioeconomic status and a greater frequency of maternal and fetal pathologies among provider-initiated preterm births can be explained by the more advanced age of these women, and a greater history of prior preterm births and cesarean sections, which probably leads practitioners to opt for expedited birth through an earlier intervention. For those women, who receive mainly private healthcare at childbirth, it appear that any potential risk condition become the motive to perform caesarean sections, despite some recent government effort to avoid it. The association of provider-initiated preterm birth with all of the investigated maternal pathologies may argue that there is a need for better national implementation of clinical protocols for appropriate indication of provider-initiated birth, with possible considerations such as waiting until term or after 39 weeks [8]. The association of provider-initiated preterm birth with prior cesarean section is also important, given the high frequency of prior cesarean section in women with more than one child (55 %) and the high rate of repeat cesarean delivery, at 80 and 98 % among women receiving public and private healthcare at childbirth, respectively [11]. Holland et al. [7]. examined provider-initiated preterm birth in a cohort of late preterm infants in the US and concluded that 80 % of this burden of prematurity could have been avoided. The preventable cases were more likely to be insured and cared for by practitioners without academic ties who scheduled the cesarean section. Other US authors have estimated the rate of unnecessary provider-initiated prematurity at 50 % [28]. In our study, it was not possible to evaluate the appropriateness of these planned interruptions in gestation. If we take as a basis studies by Bannerman et al. [28] and Holland et al. [7], however, we estimate that a reduction of between 50,000 and 80,000 late preterm births might be achieved in Brazil.

Another important fact to highlight was the lack of induction of labor in provider-initiated preterm deliveries, despite the evidence of the benefits of labor for neonatal extrauterine life and colonization with maternal microbiota, which could attenuate the disadvantages facing preterm neonates [29].

The role of infection in spontaneous/pPROM preterm birth underscores the importance of quality prenatal care. Many authors point to the importance of preventive measures during gestation to minimize premature birth. We recommend the early initiation of prenatal care, especially among adolescents, the most vulnerable group. Domingues et al. [30] found that late entry into prenatal care often makes adequate clinical care impossible because healthcare systems tend to follow the same established routine in these cases as for women who began prenatal care early, without a strategy that would counteract the delay and guarantee access to all effective interventions with the minimum of follow-up time.

Late prematurity was found at high levels in our study and represents three-quarters of all preterm births in Brazil. Given current knowledge about the crucial importance of gestational weeks 34 through 36 to the development of the neonate and the risks arising from late preterm birth [5, 31, 32] this should be a focus of public health policy. Immunological and pulmonary maturation occur during that period, and late prematurity therefore increases the risk of respiratory morbidity, longer hospital stays, neonatal ICU admission and death, as well as re-hospitalization, largely because of difficulties with breastfeeding and higher rates of neonatal jaundice and infections [1]. Adverse effects on cerebral development may underlie the neurological complications described in the short term, such as inability to effectively coordinate the movements necessary for suckling, swallowing, and breathing, and, in the longer term, delayed psychomotor development and lower school performance [2, 20, 33].

Khan et al. [23] analyzed the annual costs to society of late premature infants in the first 2 years of life, compared with term infants in the UK. They estimated that the financial burden per child was nearly 2,000 GBP over this period and argued for efforts to diminish this strain on the health system. In the USA, discussions about the projected fiscal toll of prematurity have identified the need for mitigation measures [4, 23]. Unfortunately, we are not aware of any studies that estimate the cost of prematurity in Brazil.

## Conclusion

The high rate of prematurity in Brazil may be attributed to a high proportion of provider- initiated births, especially among women receiving private healthcare at childbirth. Its association with prior cesarean deliveries and all of the studied maternal/fetal pathologies suggest that the reduction of prematurity may be possible by supporting the postponement of intervention in cases without clear evidence of maternal-fetal benefit. The association of spontaneous/pPROM prematurity with socially-disadvantaged groups confirms that the reduction of social and health inequality should continue to be a national priority.

## Abbreviations

BMI: Body mass index
GA: gestational age
IUGR: intrauterine growth restriction
pPROM: preterm premature rupture of membranes
